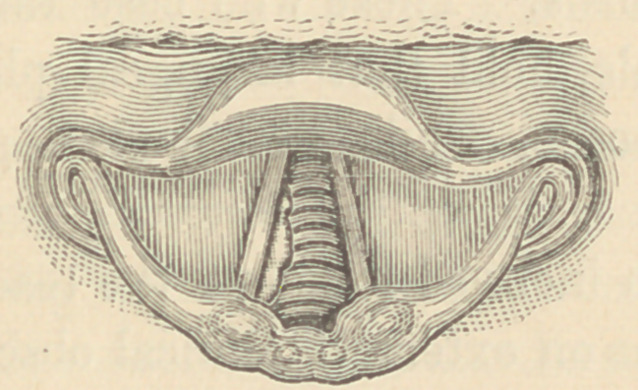# Laryngeal Tumors and Tuberculous Laryngitis

**Published:** 1879-07

**Authors:** E. Fletcher Ingals

**Affiliations:** Lecturer on Diseases of the Chest and Physical Diagnosis, and on Laryngology in the Post Graduate Course, Rush Medical College


CHICAGO MEDICAL

JOURNAL & EXAMINER.

VOL. XXXIX.—JULY, 1879.—No. 1.

[In these pages dimension and weight are expressed in terms of the Metric System, and temperature in degrees of the Centigrade Scale.]

### Original Lectures.

### ARTICLE I.

LARYNGEAL TUMORS AND TUBERCULOUS LARYNGITIS. Clinical Lecture. By E. FLETCHER INGALS, M.D., Lecturer on Diseases of the Chest and Physical Diagnosis, and on Laryngology in the Post Graduate Course, Rush Medical College.

The first patient I show you this morning was recently sent to me by my friend Dr. R. L. Leonard, of this city, on account of some difficulty experienced in breathing and speaking.

The patient, Mr. S. P., is sixty-nine years of age, is a man of good habits, and has enjoyed the best of health until two months since, with the exception of some hoarseness and cough.

He complains that for the last eight weeks he has been suffering from "some form of asthma," which has caused difficulty of breathing, especially on exertion, and has several times excited paroxysms of suffocation. He has suffered no pain and, so far as we can learn, has had no constitutional symptoms. Twenty-two months since, he first noticed impairment of function in the vocal organs, as indicated by hoarseness coming on after speaking for a short time; this gradually increased until he came under my observation, when he could only speak in a low whisper, and was obliged to pause frequently to take breath.

The slightest exertion greatly increased his dyspnœa, and to-day he tells us that he finds it impossible to sleep on his left side, because of the difficulty in breathing in that position. He has had only a little cough, though he states that frequently he desires to cough but cannot. He expectorates a small quantity of frothy mucus.

Until recently the patient's digestive organs seem to have been in perfect condition, but since the dyspnœa has been so great, his appetite has failed.

The pharynx is normal, the lungs yield no sign of disease and we naturally conclude that the cause of his trouble must be in the larynx.

At my first examination of this patient, I discovered a morbid growth, partly filling the glottis. Subsequent careful examinations, after the irritability of the throat had in a measure been overcome, revealed a large tumor filling about four-fifths of the chink of the glottis. This was of a pinkish white color, lobulated in form and seemed to be attached by a broad base to the right vocal cord and ventricular band. The right cord and the anterior third of the left were entirely hidden from view. The laryngoscopic appearance is well illustrated by this drawing.

The tumor slightly changes its position at times, so as to diminish or increase the dyspnœa, and this, together with the collection of tenacious mucus in the larynx and possibly some spasm of the glottis, an occasional complication in such cases, explains the paroxysms of suffocation.

Tumors of nearly every variety known to pathologists, have been found in the larynx; the greater part of them are benign, but encephaloid and epithelial cancers are not uncommon. Of the benign growths the papillomata constitute nearly three-fourths; fibromata stand next in frequency; following them fibro-cellular tumors, and then cystic growths which are comparatively rare.

Benign growths are said to originate in simple catarrhs, syphilitic and tuberculous sore throat, the exanthemata, particularly measles, croup, diphtheria and pertussis. Morell Mackenzie states that they originate simply in hyperæmia, and that syphilis and phthisis are not predisposing causes. Dr. Cohen, in his recent work, dissents from this opinion and gives statistics which show conclusively that tumors often occur in patients affected with syphilis or phthisis and which seem to prove that they, in some cases at least, are the direct result of these dyscrasiæ; however this may be, in the patient before us we cannot suspect any other cause than simple hyperæmia.

Prof. I. N. Danforth has examined portions of this tumor microscopically and he pronounces it a mixed sarcoma, made up of round and spindle-shaped cells, of the recurrent variety which is almost sure to return within five years in a malignant and fatal form. The grave prognosis which this opinion would induce us to give, is greatly mitigated by the fact that many cases of laryngeal growths in which the histological features have been decidedly those of cancer, have been proven clinically to be of a totally opposite character.

We have here none of the characteristic thickening or ulceration of malignant growths or those resulting from syphilis or tuberculosis; the even surface and peduncle common to fibrous and fibro-cellular tumors are lacking, therefore we conclude that this is a benign papillary tumor.

Tumors of this character usually develop slowly and after attaining a certain size they may cease to grow, when, if small, they often cause no annoyance excepting that due to the impairment of the voice; but if the tumor reaches a size sufficient to interfere with respiration either by spasm or mechanical obstruction of the glottis, it becomes a source of great and increasing danger to the patient, which if not removed will ere long cause fatal suffocation.

In young children the prognosis is specially unfavorable because the larynx is so small and the patient so intractable that it is a very difficult matter to remove the tumor through the mouth, nor are the prospects good of permanent relief by tracheotomy.

In adults who will submit to proper treatment, the prognosis as regards life is favorable, though in some cases a fatal bronchitis may be induced by tracheotomy, which occasionally becomes necessary. Some times even in adults it is impossible to remove the tumor through the mouth: in such instances if the growth enlarges so as to cause dyspnœa, tracheotomy must be performed; if it should subsequently cause considerable difficulty in swallowing, it must be removed after division of the thyroid cartilage by the operation known as thyrotomy. This operation has been performed in quite a large number of cases, but it has proven fatal in about one-third of these. After tracheotomy, breathing and deglutition may be easy and still a source of danger may remain due to the necessity for prolonged wearing of the canula in the trachea. This sometimes induces necrosis of the tracheal or laryngeal cartilages, affections which are synonymous with laryngeal phthisis and which have the same unfavorable issue.

The prognosis as regards the voice is good when the tumor can be removed through the natural passages. In fifty per cent. of such cases the voice will be completely restored and in more than half of the remaining cases it will be greatly improved. To justify the most favorable prognosis regarding the voice, the opening of the larynx must at least equal the average size and the fauces must not be abnormally sensitive; the tumor should not be extremely large and it must be single and pedunculated: when the opposite conditions are present or if the tumor is very small and located on the vocal cord the voice is not likely to be perfectly recovered.

In the case before us the orifice of the larynx is small on account of the position and shape of the epiglottis, and the difficulty of introducing instruments is greatly increased by the smallness of the space between the base of the tongue and the posterior wall of the pharynx, which is partly due to the unusual prominence of the lateral incisor teeth of the lower jaw. The central incisors have been lost and the remaining teeth are so prominent that the patient finds it impossible to hold the tongue properly without causing a great deal of pain when it presses on the teeth, consequently he cannot draw it out sufficiently. As the teeth are already loose I have suggested their removal, but the patient objects and I shall not insist upon it. This tumor is large and has a broad attachment, therefore, we cannot hope for perfect recovery of the voice, but as the growth is already of a size which renders it a constant source of danger there can be no question as to the proper course of treatment.

I hope to remove this growth by the natural passages, but I shall hold myself in constant readiness to perform tracheotomy in case the paroxysms of dyspnœa become serious.

I have already had several sittings with the patient. Owing to the sensitiveness of his throat and the other obstacles already mentioned I could hardly get a glimpse of the larynx at first and could not introduce the forceps until the fourth sitting ; at that time I removed with Mackenzie's tube forceps a portion of the tumor about the size of a large pea and since then I have removed with his common laryngeal forceps nearly all of the tumor which grew from the posterior half of the cord, but the angle of my forceps was so great that I could not reach the anterior part of the larynx without pressing forward the epiglottis, which would cause instant closure of the larynx so that the forceps had to be withdrawn.

I have an instrument to-day with a sharper angle which was made for me by Messrs. Sharp & Smith, of this city; with it I expect to be able to reach that part of the tumor lying close to the base of the epiglottis.

I cannot see into the larynx with the light in this amphitheatre, but if a few of you will go with me into my operating room I will show you the tumor and method of operating. * * This simple argand burner will light the larynx very satisfactorily. With the throat mirror in position you can now see the condition I described to you in the lecture room: you will also notice some œdema of the right ventricular band which has resulted from the irritation caused by the instruments when pieces of the growth were last removed. Having warmed the forceps I now carry it behind the epiglottis and quickly down upon the tumor; this causes closure of the larynx and I withdraw the instrument, bringing a piece of the tumor between its blades. This growth is so friable that it must be removed in fragments, for I can only secure that part of it which comes between the blades. If it were of firmer texture, the whole might possibly be removed at once.

Though I have succeeded in securing portions of the tumor at nearly every attempt, yet it is not all removed ; but as the patient is becoming fatigued we must desist.

I have to-day removed the greater part of the growth, but some fragments remain which I shall get at subsequent sittings, then if any minute portions cannot be secured, they will be cauterized to complete the cure.

*Case II.*—We have here another patient who comes to us complaining of hoarseness and dyspnœa. Mrs. M. is now twenty-five years of age, has been married several years and has one child now seven years of age.

She states that her voice has been affected for three months, and that she has had a cough about two years, but has not felt perfectly well since the birth of her child.

I can find nothing in her previous history, either personal or hereditary, which would lead me to suspect her cough to be of pulmonary origin, but you will at once notice the ominously sallow skin, bright eye and flushed and sunken cheek. She has lost flesh rapidly during the past few months and is now unable from weakness to attend to her household duties.

The skin is moist and of slightly increased temperature ; the thermometer under the tongue registers 38.8° C. and her pulse averages 130 per minute.

The hoarseness which was at first only slight, now compels her to talk in a low voice, scarcely louder than a whisper. She has dyspnœa on exertion and has formerly suffered from considerable cough, but at present it does not trouble her greatly. The expectoration is small in amount and of a muco-purulent character. The tongue is clean and moist and the throat normal in appearance, excepting a marked loss of its natural redness. Her appetite is fair, deglutition is not painful; and the digestive organs seem to be acting well. The menses are regular.

This is all the information we can obtain from the simple interrogation of the patient.

We must now examine the larynx in hopes of finding there the immediate cause of her trouble.

In looking into the larynx we observe a peculiar pallor of every part excepting the epiglottis and right vocal cord. The epiglottis is of twice its natural thickness and is bent sharply upon itself toward the base of the tongue, about five millimeters from its upper free edge. Just below the point of flexure the mucus membrane stands out in two or three small whitish projections which seem to mark the edge of an ulcer, but it is impossible to see the laryngeal surface of the epiglottis distinctly.

I have been able to catch a glimpse of a dark brown or black spot near the base of the epiglottis, which seems to be five or six millimeters in diameter and which probably results from destruction of the mucous membrane and exposure of the cartilage. There is no change in the ary-epiglottic folds. The right vocal cord is congested and thickened and just beneath it we find an abnormal growth which is fairly represented in this drawing.

This growth has a greyish hue and somewhat uneven surface. It is not, properly speaking, a laryngeal tumor nor is it simply infra-glottic œdema, but it appears to consist of an elevated fold of thickened mucous membrane. It has increased considerably in size since I first saw the patient, but I think it is not growing at present.

The appearance of this larynx, though not characteristic, leads me to strongly suspect laryngeal phthisis, a diagnosis which will be rendered positive if the lungs yield evidence of tuberculosis.

Upon examining the chest we find marked dullness over the lower part of the infraclavicular and the upper part of the mammary regions on the left side with broncho-vesicular respiration ; and fine mucous and subcrepitant râles over both apices. This leaves no doubt as to the diagnosis.

It is common in such cases as this for the larynx to be affected first on the side where the pulmonary disease is most marked, but it is not in this instance.

I have already stated that some distinguished laryngoscopists hold that morbid growths in the larynx are never caused by phthisis, but Dr. Cohen has found one-third of his cases associated with this disease. In the case before us, the tumefaction below the vocal cord cannot properly be called a tumor, though probably, if left to itself, its increasing size would soon compel us to class it with the case we have just considered.

In another case of laryngeal phthisis now under my care, I find a distinct tumor, the size of a large split pea, springing from the mucous membrane covering the right arytenoid cartilage; there is tumefaction of the right ary-epiglottic fold, and the history and pulmonary signs place the diagnosis beyond question. It is not at all probable that in these cases the morbid growths are simply coincidental.

Authors are unsettled as to the etiological relations of tubercle and laryngeal phthisis. Those who base their belief on clinical experience, as a rule, hold that laryngeal phthisis is not caused by tubercular deposits, while pathologists generally teach the reverse.

Personally, I am inclined to adopt the teachings of those who found their opinions on extensive clinical observation, rather than those based on a few isolated microscopic examinations, the results of which are often doubtful to microscopists themselves.

Laryngeal phthisis is nearly always associated with pulmonary consumption, sometimes preceding the latter, but generally following in its course, as it doubtless has in the case before us. The affection is characterized by thickening and ulceration of the larynx. The thickening usually appears first in the ary-epiglottic folds and subsequently affects other parts or it may begin in the epiglottis, ventricular bands or vocal cords; the ulceration is frequently first seen on the vocal cords, but in other instances it begins on the epiglottis or some other part; usually it commences in the lower parts of the larynx and extends upward. The ulceration may extend from the mucous membrane and destroy a considerable portion of one or more of the cartilages, but in other cases the cartilages themselves are first affected and finally becoming necrosed they act as foreign bodies in causing abscesses and destruction of surrounding tissues.

The prognosis in this affection is most unfavorable; patients seldom live more than from six to eighteen months and when any considerable thickening has taken place a fatal result is almost absolutely certain. Those cases in which the epiglottis is first attacked run the most rapid course. The ulcers show no tendency to heal and very little can be hoped for from treatment excepting to mitigate suffering, slightly prolong life and perhaps stay the progress of the local affection.

In the early stage of laryngeal phthisis when there is simple hyperæmia the local applications recommended for chronic laryngitis will often be found beneficial, and later on we may expect to check the progress of the disease in some instances by the application of mineral astringents, and thus prevent the distress incident to extensive ulceration, or necrosis and exfoliation of the cartilages. When the epiglottis or the ary-epiglottic folds are the seat of the ulceration, the tissues are likely to be destroyed to such an extent as to prevent proper closure of the glottis in the act of swallowing ; as a result fluids or food escape into the larynx and give rise to severe paroxysms of cough and suffocation, which are so very distressing that patients will sometimes go for days without food or drink rather than endure the suffering almost sure to follow attempts at deglutition.

While attending to the local symptoms, we must not forget the constitutional malady which requires the same treatment as uncomplicated pulmonary consumption.

In this patient I have made slightly stimulant and astringent applications to the larynx which have arrested the growth for the time being, but the applications, whether in the form of powdered insufflations or in solutions applied by means of a camel's hair pencil, cause so much spasm of the glottis that I shall substitute for them inhalations with which I hope to check in some degree the rapid progress of the disease.

The application which has been most efficient in this case consists of one part of the sulphate of berberina to eight parts of sugar of milk, about 0.10 Gm. of which have been applied to the larynx every second or third day. I have also tried the local application of calomel, bismuth and tannin, but although they often prove beneficial in laryngitis they have done no good in this case. On account of the suffocation caused by insufflations I resorted to the application of a solution of chloride of zinc, 2.00 Gm. to 50.0 Gm. of glycerine ; but it caused spasms, quite as severe as the powders. I therefore discontinued it and the patient is now using the following prescription:

℞. Coniæ 0.25 Gm., alcoholis 12.00 Gm., olei pinus sylvestris 12.00 Gm., magnesiæ carb. levis 8.00 Gm., aquæ qs *ad.* 100.00 CC, M., triturate. S. Teaspoonful to be used in a pint of water at 65° C. for an inhalation, every fourth or fifth hour.

When I first saw this patient she had little appetite, and I ordered strychinæ sul. 0.02 Gm. and tinct. ferri chlor. 0.50 Gm. three times daily, combined with calcii chloridum 0.65 Gm. I gave the chloride of calcium because, from an extensive use of it for nearly two years, I have become convinced that it exercises a very beneficial influence on a large percentage of phthisical patients.

The results of treatment have thus far been very satisfactory, but we can hardly hope for permanent benefit.

In this case I shall enjoin out-door exercise every pleasant day, and if it were possible I should send her to a climate where the air is uniformly warm and dry.

This treatment will undoubtedly benefit the patient, but I fear the progress of the disease will be rapid and her history will be completed within three or four months.

---

A LITTLE book has been sent to each practising physician and surgeon in the United States, whose name and address are known to the Census Office, with the request that each will keep therein a record of all deaths occurring within his practice during the year June 1, 1879, to May 31, 1880, and will return the register at the close of the year to the Census Office. It is hoped that this effort to improve the Vital Statistics of the United States will meet with general approbation and receive the countenance and support of the profession.

## Figures and Tables

**Figure f1:**
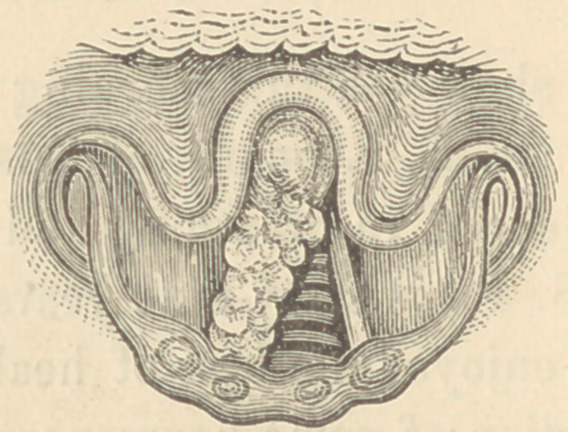


**Figure f2:**